# Complete Genome Sequence of an Aquaculture-Associated Phage, FL-1, Infecting *Flavobacterium* spp.

**DOI:** 10.1128/genomeA.00014-17

**Published:** 2017-06-08

**Authors:** Elina Laanto, Janne J. Ravantti, Lotta-Riina Sundberg

**Affiliations:** a Department of Biological and Environmental Science and Nanoscience Center, Centre of Excellence in Biological Interactions, University of Jyvaskyla, Jyvaskyla, Finland; b Department of Biosciences and Institute of Biotechnology, University of Helsinki, Helsinki, Finland

## Abstract

FL-1, a myophage of *Flavobacterium*, was found to have a 53-kb genome with 87 putative coding sequences.

## GENOME ANNOUNCEMENT

*Flavobacterium* spp. are abundant and widespread in freshwater environments (1, 2). Despite the prevalence of the members in this genus, the phages infecting *Flavobacterium* spp. are less known. So far, most of the phages studied in detail infect fish pathogenic members of the genus, such as *F. psychrophilum* (3).

The phage FL-1 and its *Flavobacterium* sp. host strain B183 were previously isolated from a water sample from a fish farm in Central Finland. Transmission electron microscopy revealed FL-1 to be a member of the family *Myoviridae* (4). In addition to the isolation host, FL-1 also infects multiple *Flavobacterium* sp. isolates and strains of the fish pathogen *F. columnare*, the causative agent of columnaris disease in fish (5).

DNA was extracted from FL-1 phage lysate, as described by Santos (6) with slight modifications as described earlier (7). DNA was sequenced on two platforms—Ion Torrent PMG with a 100-bp kit and commercially with Roche 454 (LGC Genomics)—and the data were combined (neither method resulted in a whole-genome sequence). Analyses were done using GS De Novo Assembler version 2.9 (Roche 454 Life Sciences), which uses an overlap layout consensus methodology. The average coverage was 11×, and the inferred read error rate was 0.94%. The assembly resulted in three scaffolds that were combined using Sanger sequencing. Glimmer and GeneMark were used for predicting the open reading frames (ORFs) using Geneious version 7.1 (Biomatters Ltd.), with possible functions predicted using BLASTp (8). tRNAscan-SE (9) was used to search for putative genes coding for tRNAs, but none were detected. The genome of FL-1 comprises 53,088 bp with a G+C content of 32.4%. Of the 87 predicted ORFs (ranging from 120 to 2,043 bp in length), only two were leftward oriented. Start codon usage was 94% for ATG, 3% for TTG as an initiation codon, and 2% for GTG. Putative functions were assigned for only six of the predicted coding sequences (CDSs), including a terminase, a portal protein, a phage lysin, and two tail proteins. The remaining 81 ORFs were assigned as hypothetical proteins. Primer walking confirmed the ends of the genome. Interestingly, only five of the CDSs had a recognizable Shine-Dalgarno sequence. However, the ribosomal binding site consensus sequence, TAAAA, has been proposed for environmental *F. hibernum* (10). Indeed, this sequence was also found in the upstream (20 bp) sequence for 34 of the CDSs and a TAAA sequence for 24 of the CDSs.

BLASTp analysis of the FL-1 predicted ORFs found several hits to the *Cellulophaga* phage phiSM and *F. columnare* phage FCL-2 genomes (21 and 30, respectively). An alignment of these three genomes using Mauve (11) revealed FL-1’s relationship to both FCL-2 and phiSM, as all shared a conserved region that includes the putative structural proteins, indicating shared synteny.

### Accession number(s).

The complete genome of *Flavobacterium* sp. phage FL-1 was deposited in GenBank under the accession number KY421186.
